# Diversity in Expression of Phosphorus (P) Responsive Genes in *Cucumis melo* L

**DOI:** 10.1371/journal.pone.0035387

**Published:** 2012-04-19

**Authors:** Ana Fita, Helen C. Bowen, Rory M. Hayden, Fernando Nuez, Belén Picó, John P. Hammond

**Affiliations:** 1 Centro de Conservación y Mejora de la Agrodiversidad Valenciana, Universitat Politècnica de València, Valencia, Spain; 2 Warwick HRI, University of Warwick, Wellesbourne, Warwick, United Kingdom; University of Michigan, United States of America

## Abstract

**Background:**

Phosphorus (P) is a major limiting nutrient for plant growth in many soils. Studies in model species have identified genes involved in plant adaptations to low soil P availability. However, little information is available on the genetic bases of these adaptations in vegetable crops. In this respect, sequence data for melon now makes it possible to identify melon orthologues of candidate P responsive genes, and the expression of these genes can be used to explain the diversity in the root system adaptation to low P availability, recently observed in this species.

**Methodology and Findings:**

Transcriptional responses to P starvation were studied in nine diverse melon accessions by comparing the expression of eight candidate genes (*Cm-PAP10.1*, *Cm-PAP10.2*, *Cm-RNS1*, *Cm-PPCK1*, *Cm-transferase*, *Cm-SQD1*, *Cm-DGD1* and *Cm-SPX2*) under P replete and P starved conditions. Differences among melon accessions were observed in response to P starvation, including differences in plant morphology, P uptake, P use efficiency (PUE) and gene expression. All studied genes were up regulated under P starvation conditions. Differences in the expression of genes involved in P mobilization and remobilization (*Cm-PAP10.1, Cm-PAP10.2 and Cm-RNS1*) under P starvation conditions explained part of the differences in P uptake and PUE among melon accessions. The levels of expression of the other studied genes were diverse among melon accessions, but contributed less to the phenotypical response of the accessions.

**Conclusions:**

This is the first time that these genes have been described in the context of P starvation responses in melon. There exists significant diversity in gene expression levels and P use efficiency among melon accessions as well as significant correlations between gene expression levels and phenotypical measurements.

## Introduction

Phosphorus (P) is major limiting nutrient for plant growth [1], [2]. Therefore, crops are frequently supplied with inorganic phosphate (Pi) fertilizers to maintain yields and quality. However, the application of Pi fertilizers is problematic for both the intensive and extensive agriculture of the developed and developing countries, respectively. In intensive agriculture, excess of soluble inorganic Pi fertilizers is leading to eutrophication and hypoxia of water bodies [3]. In extensive agriculture in the tropics and subtropics, chemically imbalanced soils reduce the availability of P to crops and a lack of infrastructure and purchasing power for fertilizers compound these issues [4]. In addition, since over 85% of mined P is used in food production [5] and consumption of this non-renewable resource will lead to peak phosphorus production (akin to peak oil; [6], [7]), there will be increasing pressures on Pi fertilizer availabilities and costs in the future. These pressures will be exacerbated by increasing demand on food production systems as the human population increases and by fluctuation in oil prices [7]. Sustainable management of P in agriculture requires developing crops with enhanced P efficiency and management schemes that increase soil Pi availability [8]. Plants have developed adaptive strategies to cope with low soil P availability. These include: i) improvement of Pi-utilization efficiency by enhancing Pi internal remobilization, transport and metabolism, and ii) improvement of Pi-acquisition efficiency by modifying root systems and mobilizing Pi from the soil [1], [9].

Over the past two decades, extensive studies of the response to P starvation using *Arabidopsis thaliana* as a readout phenotype have contributed markedly to the understanding of P signaling and response pathways. In addition, transcriptional profiling of *A. thaliana*, other globally important crops such as *Oryza sativa* (rice), *Lupinus albus* (white lupin), *Phaseolus vulgaris* (common bean), *Brassica rapa* (Chinese cabbage), *Solanum tuberosum* (potato) and *Zea mays* (maize) have extend our knowledge and revealed the complexity of the network of genes necessary for plants to adapt to low soil P availability [10]–[18]. These studies have revealed a series genes involved in the adaptations to low P, mainly through the regulation of P acquisition, intern remobilization, change in metabolism, and signal transduction [9]. For instance, expression of genes encoding purple acid phosphatases (PAPs) and ribonucleases (RNS) are generally up-regulated under Pi starvation. PAPs are involved in releasing Pi from organic sources, both internally and externally, for efficient transport and subsequent use. They are important in the mobilization of the organic P in soil for root absorption, remobilization of organic P in senescing organs, storage tissues, and intracellular compartments [19], [20]. In addition, several studies in arabidopsis, rice and white lupin have demonstrated how Pi starvation affects carbon metabolism [11], [21], [22], resulting in the accumulation of starch in the leaves and increasing anthocyanin production to protect the photosynthetic machinery. Phosphate starvation also provokes modifications in the lipid composition of plant membranes, including a decrease of phospholipids and an increase of non-phosphorous lipids. Consequently, a typical transcriptional response to Pi starvation is the up regulation of enzymes involved in sulfolipid and galactolipid synthesis [23]–[26]. In addition, the transcripts and activity of Pi transporters are increased to optimize uptake and remobilization of Pi in Pi-deficient plants [10]–[12], [14], [18], [27].

Melon (*Cucumis melo* L.) ranks as the 9th most cultivated horticultural crop in terms of total world production. This species belongs to the botanical family Cucurbitaceae, commonly known as cucurbits. It is an important crop in tropical and subtropical areas, many of which have P-deficient soils. The species is considered to be divided into two subspecies, ssp. *melo* and ssp. *agrestis*, each one with several botanical varieties that display a rich morphological diversity [28]. Botanical varieties belonging to the ssp. *agrestis* are wild or exotic types found in Africa and eastern Asia, from India to Japan, and those belonging to the ssp. *melo* are mainly cultivated types found from India to Europe and in the Americas. The main ssp. *agrestis* varieties are *conomon* (Thunberg) Makino, *momordica* (Roxburgh) Duthie & Fuller, and the main ssp. *melo* varieties are *dudaim* (L.) Naudin, *flexuosus* (L.) Naudin, *cantalupensis* Naudin, and *inodorus* Jacquin, with *cantalupensis* and *inodorus* containing most of the commercial varieties [28]. Variability in root morphology and architecture has been described within this species, especially between varieties of both subspecies [29], [30]. The relationships between root architecture and response to Pi starvation has also been studied, showing a high variability in the acquisition and use of Pi among melon varieties [31]. However, to our knowledge, there is no information available on the genes involved in the Pi starvation response in melon.

The availability of genetic and genomic resources allows the use of the arabidopsis vast information on P homeostasis related genes on other species [32]. Genomic resources for melon have increased significantly in recent years. These tools include a complete transcriptome, with 53,252 accurately annotated unigenes, assembled from a collection of 125,908 and 689,054 expressed sequence tags (ESTs) [33], [34] and 454 sequencing methodologies from a number of accessions belonging to both subspecies [35]. Whole genome sequence of this crop is also in progress [36]. These recent advances make it possible to identify melon orthologues of candidate Pi responsive genes described in other species.

Here we report, diversity in the expression of Pi starvation responsive genes in nine different melon varieties with contrasting phenotypic responses to Pi starvation [31]. Representatives of the two subspecies of melon, the main commercial groups and the exotic types mostly used in melon breeding are included.

## Results

### Biomass allocation and PUE traits vary significantly between melon accessions

The biomass allocation and PUE traits were analyzed under P replete (control, C) and P starving (NoP) conditions in a genetically diverse set of melon accessions: three accessions belonging to ssp. *agrestis* (chi-SC and ma-YP of var. *conomon*, and mo-kha of var. *momordica*) and six accessions belonging to ssp. *melo* (cha-PI and flex-Ac of var. *flexuosus* and four varieties of the main commercial groups, re-Du of var. *reticulatus*, ca-NC of *var. cantalupensis* and In-Ps and In-Am of var. *inodorus* (Table 1).

**Table 1:** List of accessions studied and their origin.

Botanical variety^a^	Botanic group^b^	Name^c^	Origin
***C. melo ssp.agrestis***			
*conomon*			
chi-SC	*chinensis*	Songwhan Charmi (PI161375)	Korea
ma-YP	*makuwa like*	Yamato Purinsu	Japan
*momordica*			
mo-Kha	*momordica*	Kharbuja	India
***C. melo ssp. melo***			
*flexuosus*			
cha-PI	*chate*	PI 490388	Mali
flex-Ac	*flexuosus*	Acuk (PI 167057)	Turkey
*cantalupensis*			
re-Du	*reticulatus*	Dulce	USA
ca-NC	*cantalupensis*	Noir des Carmes	France
*inodor*us			
in-PS	*inodorus*	Piel de sapo	Spain
in-Am	*inodorus*	Amarillo	Spain

a Tentative classification according to Munger and Robinson [52].

b Tentative classification according to Pitrat [28].

c Accessions were kindly supplied by COMAV-UPV, ARS-GRIN-USDA and IPK-Gatersleben germplasm banks.

**Figure 1:**
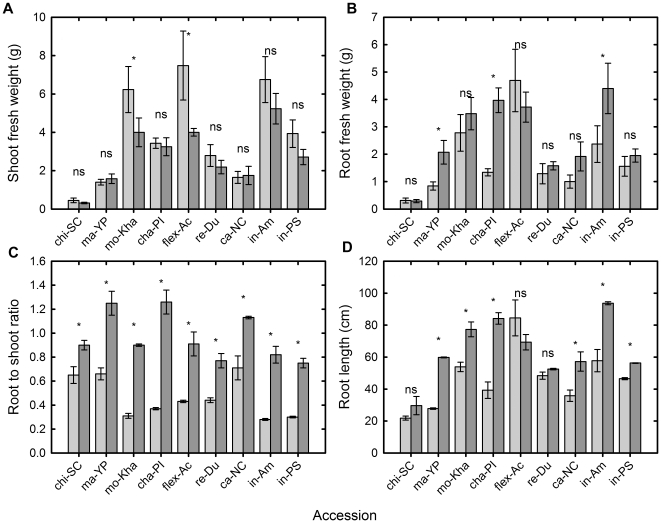
Biomass, root to shoot ratio and root length for nine melon accessions grown hydroponically under P replete and P starved conditions. Mean (a) shoot fresh weight, (b) root fresh weight, (c) root to shoot ratio and (d) root length for melon accessions grown hydroponically with a full nutrient solution (Control, light grey bars) or nutrient solution containing no phosphate (NoP, dark grey) for 21 d. Each bar represents the mean ± se (n = 9). ^*^ The mean value in NoP treatment was significantly different at *P*<0.05 from the mean value of the same accession in Control treatment. ^ns^ no significant difference.

There were significant differences in root fresh weight (RFW), shoot fresh weight (SFW), root to shoot ratio (RSRa) and root length (RL) among accessions within the control treatment (Figure 1). Accessions belonging to *conomon* group of ssp. *agrestis*, chi-SC and ma-YP, in general had low SFW and RFW, and high RSRa, which was not the case of mo-Kha, which also belongs to ssp. *agrestis*. From the *melo* ssp., flex-Ac had the highest SFW and RFW, followed by in-Am. Cha-PI, in-PS and re-Du had intermediate values for SFW and RFW, whereas ca-NC displayed morphological traits similar to ma-YP. The lack of P did not produced big reductions in SFW, only mo-kha and flex-Ac showed a significant reduction in SFW between C and NoP treatments (Figure 1A). Despite the tendency of increasing the RFW under NoP condition, only ma-YP, cha-PI and in-Am showed a significant increase in their RFW between C and NoP treatments (Figure 1B). However, the increase of RSRa and RL was significant for all the accessions (except for RL in chi-SC, flex-Ac and re-Du; Figure 1C and D). Although the treatment period was insufficient to significantly reduce SFW, it is clear that the plants are responding to the treatment by altering resource allocation and increasing RSRa. It is interesting to note that flex-Ac had the longest root length in the control treatment, and despite being the only accession to reduce its RL in the NoP treatment still ranked fourth for RL, following in-Am, mo-Kha, and cha-PI, in the NoP treatment (Figure 1D).

**Figure 2:**
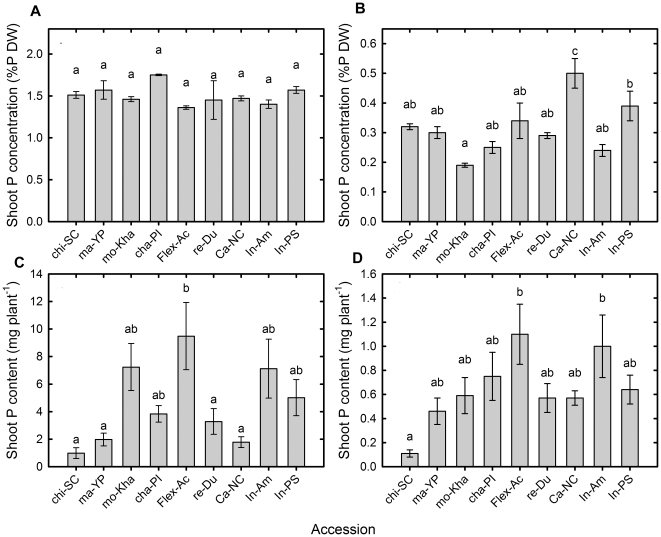
Phosphorus content in shoots of nine melon accessions grown hydroponically under P replete and P starved conditions. Mean (a,b) shoot P concentration and (c,d) shoot P content for melon accessions grown hydroponically with a full nutrient solution (Control, a,c) or nutrient solution containing no phosphate (NoP, b,d) for 21 d. Each bar represents the mean ± se (n = 9). Bars with the same letter are not significantly different at *P*<0.05 by Newmans Keuls multiple range test.

There were no significant differences among accessions for shoot P concentration (Shoot-[P], %) in control conditions (Figure 2A), but significant differences were observed for shoot P content (Shoot-P, mg P plant^−1^), where flex-Ac had the highest Shoot-P (9.48 mg P plant^−1^; Figure 2B), mo-kha, in-Am, in-PS and cha-PI where intermediate, ranging from 3.28–7.23 mg P plant^−1^, and chi-Sc, ma-YP and ca-NC had the lowest values (0.98 to 1.97 mg P plant^−1^). Under NoP conditions, the lowest Shoot-[P] was observed in mo-kha (0.19 P%) and the highest was observed in ca-NC (0.5 P%), followed by in-PS (0.39 P %; Figure 2C). For Shoot-P in NoP conditions, flex-Ac and in-Am had the highest values (1.1 and 1 mg P plant^−1^, respectively) and chi-SC again had the lowest value (0.11 mg P plant^−1^; Figure 2D). Taking into account that there was no P in the NoP treatment, these differences in Shoot-P should be considered as differences in the accumulation of P on the genotypes seeds (all seeds were produced under the same conditions in our greenhouses).

**Figure 3:**
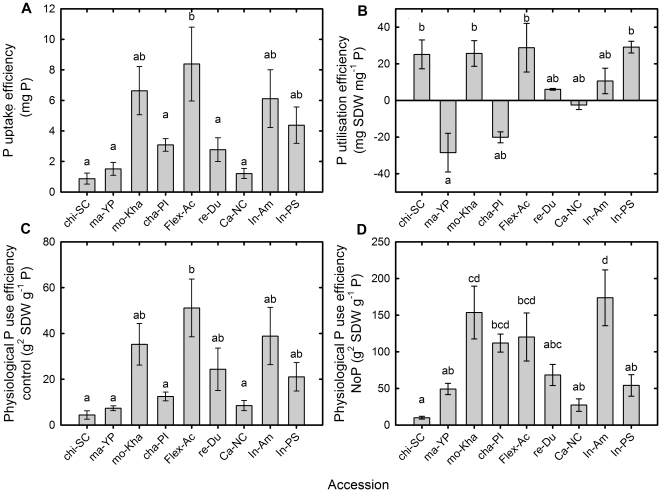
Phosphorus use and uptake measures of nine melon accessions grown hydroponically under P replete and P starved conditions. Mean (a) Phosphorus (P) uptake efficiency, (b) P utilization efficiency, (c) physiological P use efficiency for melon accessions grown hydroponically with a full nutrient solution, and (d) physiological P use efficiency for melon accessions grown hydroponically with a nutrient solution containing no phosphate for 21 d. Each bar represents the mean ± se (n = 9). Bars with the same letter are not significantly different at *P*<0.05 by Newmans Keuls multiple range test.

There were significant differences for P use efficiency traits among the melon accessions (Figure 3). Chi-SC, ma-YP and ca-NC had low values for PUpE and PPUE, whereas their values for PUtE were variable, including negative values for ma-YP and a high positive value for Chi-SC. Within the ssp. *agrestis*, mo-Kha had significantly different values for PUpE and PPUE compared with other *agrestis* accessions chi-SC and ma-YP (Figure 3). Within the ssp. *melo*, Flex-Ac had the highest values for PUpE, PUtE and PPUE under control conditions and the second highest for PPUE under the NoP treatment. The *inodorus* accessions also had moderate to high values for the different measures of PUE, with in-Am having the highest value for PPUE under the NoP treatment (Figure 3D). Re-Du and cha-PI, had intermediate values for PUE traits, between *inodorus* and *conomon* groups, cha-PI also had a negative value for PUtE as consequence of its higher SDW under P starvation.

### Expression of genes involved in response to P starvation

The expression of genes involved in plant responses to P starvation (phosphorus uptake and mobilization, carbon and secondary metabolism, alteration of membrane lipids composition and P transport) were analyzed under control and NoP conditions in a genetically diverse set of melon accessions.

### Phosphate mobilization and re-mobilization

**Figure 4:**
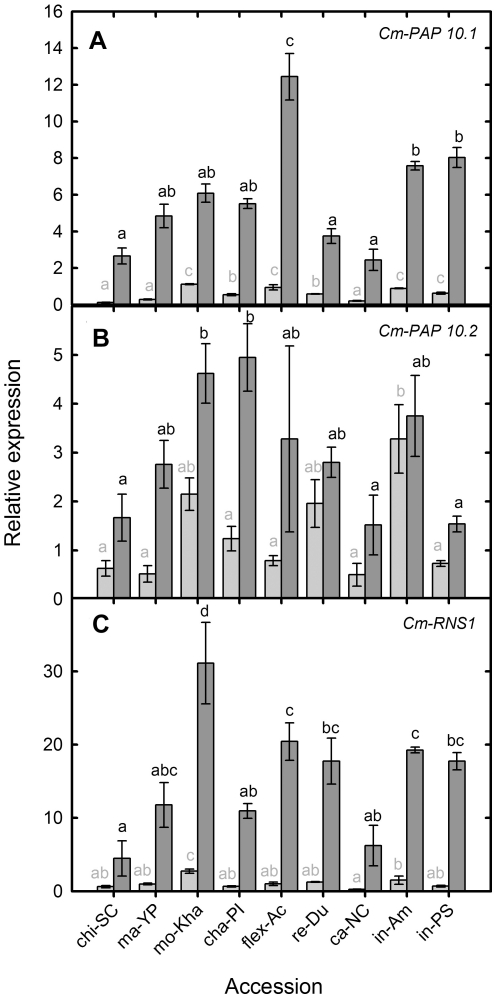
Root transcript abundance for *Cm-PAP10.1*, *Cm-PAP10.2*, *Cm-RNS1* in nine melon accessions under P replete and P starved conditions. Relative transcript abundance for (a) *Cm-PAP10.1* [MU46092], (b) *Cm-PAP10.2* [MU50216], (c) *Cm-RNS1* [MU47003] in different melon accessions grown hydroponically with a full nutrient solution (Control, light grey) or nutrient solution containing no phosphate (NoP, dark grey) for 21 d. Transcript abundance was measured using quantitative PCR (Q-PCR) and expressed relative to that of the housekeeping gene (*Cm-Ubiquitin*). Grey bars with the same grey letter are not significant different at *P*<0.05 by Newmans Keuls multiple range test.

Of the two putative purple acid phosphatases profiled (*Cm-PAP10.1* and *Cm-PAP10.2*, ICUGI unigenes MU46092 and MU50216), the relative expression of *Cm-PAP10.1* was lower than that of *Cm-PAP10.2* in control conditions (Figure 4A and B). Under NoP conditions both genes were induced, with *Cm-PAP10.1* having a greater fold change induction in expression than *Cm-PAP10.2*, with the former induced between 5 to 17-fold, and the latter induced between 1.67 to 6.71-fold (Figure 4B). The relative expression of these two genes in the studied accessions was variable. Chi-SC, re-Du and ca-NC showed low to moderate relative expression levels in both treatments and genes. Under NoP conditions, mo-Kha, cha-PI, had moderate expression of *Cm-PAP10.1*, but high expression of *Cm-PAP10.2*. In contrast, in-Am and in-PS had high expression of *Cm-PAP10.1* and moderate to low expression of *Cm-PAP10.2* under NoP conditions. Flex-Ac showed the highest relative expression of *Cm-PAP10.1* and a moderate expression of *Cm-PAP10.2* in NoP conditions. Interestingly, despite the low expression of these genes under NoP conditions for chi-SC and ma-YP accessions, they showed a greater induction in expression from control to NoP conditions (Figure 4A and B).

*Cm-RNS1* (ICUGI unigen MU47003), which encodes a ribonuclease, was also induced under NoP conditions (Figure 4C). The relative basal expression levels in control conditions were close to one, with a significant increase under NoP conditions. The change of expression was similar among accessions except for chi-SC which showed the lowest increase (7-foldhigher expression in NoP than in the control) and in-PS which showed the highest one (26-fold). The accession with the highest relative expression under NoP condition was mo-kha (31.12) followed by flex-Ac and in-Am (Figure 4C).

The expression of these targets putatively involved in P mobilization/remobilization correlated with changes in root structure and measures of PUE (Table 2). The expression of *Cm-PAP10.1* was positively correlated with SFW, RFW and RL in control and NoP treatments, suggesting that higher levels of gene expression are related with higher plant size no matter the treatment. Whereas *Cm-PAP10.2* and *Cm-RNS1* relative expressions showed significant correlations with SFW, RFW and RL, under NoP treatments, indicating that these genes may be associated with improved biomass allocations under NoP conditions. RSRa was negatively correlated with the relative expression of *Cm-PAP10.1* and *Cm-PAP10.2* in control conditions, and accessions with high RSRa, such as chi-SC, ma-YP and ca-NC, had lower basal levels of expression of these genes.

**Table 2:** Correlation among phenotypical traits and gene expression.

	*Cm-PAP10.1* a	*Cm-PAPP10.2*	*Cm-RNS1*	*Cm-PPCK1*	*Cm-transferase*	*Cm-DGD1*	*Cm-SQD1*	*Cm-PHO1*
SFW								
C	0.73***	0.31	0.23	0.29	−039*	−0.40*	0.11	0.35
NoP	0.51*	0.31	0.65***	0.15	−0.12	−0.36	0.15	0.26
RFW								
C	0.65***	0.13	0.13	0.12	−0.43*	−0.46*	0.03	0.23
NoP	0.52*	0.44*	0.53***	−0.03	−0.17	0.32	0.04	0.05
RSRa								
C	−0.67***	−0.60**	−0.43	−0.68***	−0.05	0.32	−0.28	−0.56*
NoP	−0.08	0.26	−0.24	−0.54***	−0.05	0.12	−0.55	−0.63*
RL								
C	0.56***	0.19	0.12	0.21	−0.44	−0.54	0.09	0.26
NoP	0.43*	0.56*	0.43	−0.11	−0.07	−0.34	−0.13	0.00
P%								
C	−0.32	−0.30	−0.09	−0.19	0.06	0.11	0.10	−0.11
NoP	−0.31	−0.77***	−0.54***	0.28	0.02	−0.10	−0.37	−0.46
P								
C	0.74***	0.26	0.15	0.26	−0.44**	−0.40	−0.1	0.35
NoP	0.37	−0.12	0.32	0.08	−0.09	−0.52*	−0.02	0.13
PUpE	−0.52***	−0.07	0.61***	−0.22	0.35*	0.22	0.23	−0.32
PUtE	−0.37	−0.26	0.37	0.15	0.37*	0.21*	0.34*	−0.24
PPUE								
C	0.62***	0.36	0.15	0.28	−0.38*	−0.39*	0.05	0.32
NoP	0.34	0.51**	0.68***	0.10	−0.06	−0.21	0.15	0.25

Shoot fresh weight (SFW), root fresh weight (RFW), root to shoot ratio (RSRa), root length (RL), P concentration ([P]), shoot P, P uptake efficiency (PUpE), P use efficiency (PUtE) and physiological use efficiency (PPUE) with the relative expression of the studied genes.

a Each data is the Pearson correlation of the studied trait with the normalized value of expression of each gene. In PUpE and PUtE the correlation is calculated with the fold-change of expression of the gene from NoP to control treatment.

*,**,*** , are significant at P<0.05,0.01 and 0.001 respectively. ^ns^ non-significant.

The expression of *Cm-PAP10.1* was also highly positively correlated with shoot P content and PPUE under control conditions but not under NoP conditions. *Cm-PAP10.1* was less expressed in those accessions with small vines and roots in control conditions. These poorly performing accessions had high increases in the expression of the gene when passing form control to NoP but not great PUpE resulting in a negative correlation between PUpE and *Cm-PAP10.1* induction. Interestingly *Cm-PAP10.2* had different response; it has a negative correlation with [P] and a positive correlation with PPUE under NoP conditions. Therefore, it seems that *Cm-PAP10.1* is correlated with better performance in control conditions and *Cm-PAP10.2* is correlated with higher performance under NoP conditions. *Cm-RNS1* showed similar correlations with other studied traits to *Cm-PAP10.2*, but in this case *Cm-RNS1* was also positively correlated with PUpE, therefore it may contribute to increase uptake in presence of P but also contribute to perform better under NoP conditions.

### Carbon and secondary metabolism

**Figure 5:**
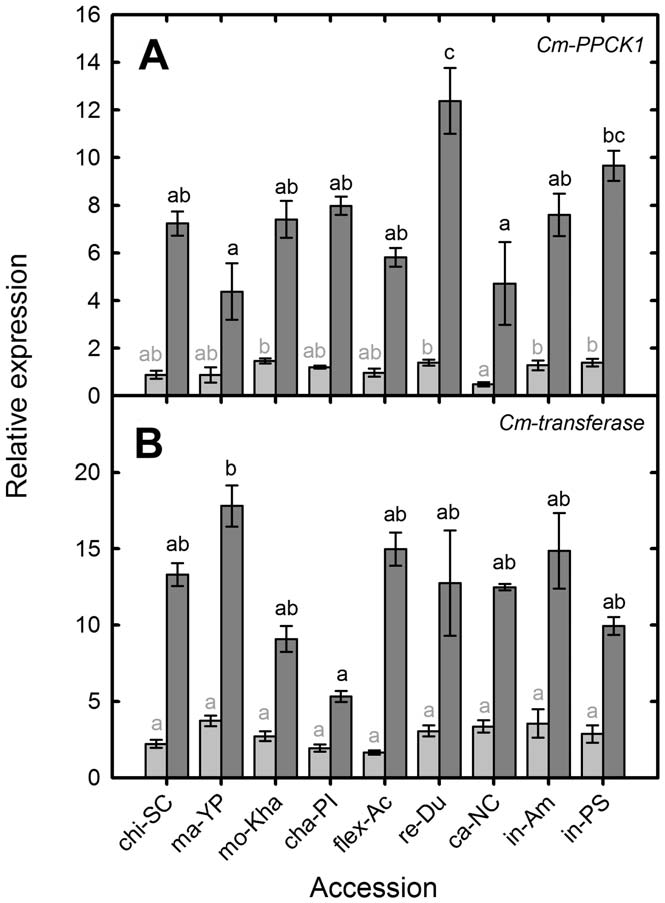
Root transcript abundance for *Cm-PPCK*, *Cm-transferase* in nine diverse melon accessions under P replete and P starved conditions. Relative transcript abundance for (a) *Cm-PPCK1* [MU52466], (b) *Cm-transferase* [MU47437] in different melon accessions grown hydroponically with a full nutrient solution (Control, light grey) or nutrient solution containing no phosphate (NoP, dark grey) for 21 d. Transcript abundance was measured using quantitative PCR (Q-PCR) and expressed relative to that of the housekeeping gene (*Cm-Ubiquitin*). Grey bars with the same grey letter are not significant different at *P*<0.05 by Newmans Keuls multiple range test. Dark grey bars with the same letter are not significant different at *P*<0.05 by Newmans Keuls multiple range test.

The expression of genes known to be involved in carbon and secondary metabolism, were also measured in all nine accessions. These included a phosphoenol pyruvate kinase, *Cm-PPCK1*, and an anthocyanin 5-aromatic acetyltransferase, *Cm-transferase*, (ICUGI unigenes MU52466 and MU47437). Under NoP conditions, the expression of CmPPCK1 increased on average 7-fold across accessions compared with the control treatment (Figure 5A). Ma-YP and ca-NC showed lower levels of expression under NoP (4.38 and 4.72 respectively) and re-Du had the highest level of expression (12.38) followed by in-PS. Under the NoP condition the expression of *Cm-transferase* increased on average 7-fold across accessions compared with the control treatment (Figure 5B). There were no large differences in relative expression among accessions under NoP treatment, except for ma-YP (17.81) with the greatest expression, and cha-PI with the lowest (5.33). The highest induction of expression was achieved by flex-AC with a 9-fold increase in expression (Figure 5B).

The only significant correlation between the relative expressions of *Cm-PPCK* and phenotypical traits was a negative correlation with RSRa in both treatments. The expression of *Cm-transferase* was negatively correlated with SFW, RFW, P content and PPUE, under control conditions, indicating that higher expression of this gene under control conditions was associated with low PUE. However, the increased expression of *Cm-transferase* from control to NoP conditions was positively correlated with PUpE and PUtE, suggesting that this gene might be associated with changes in the efficiencies of P uptake and use under NoP conditions.

### Alteration membrane lipid composition and metabolism

**Figure 6:**
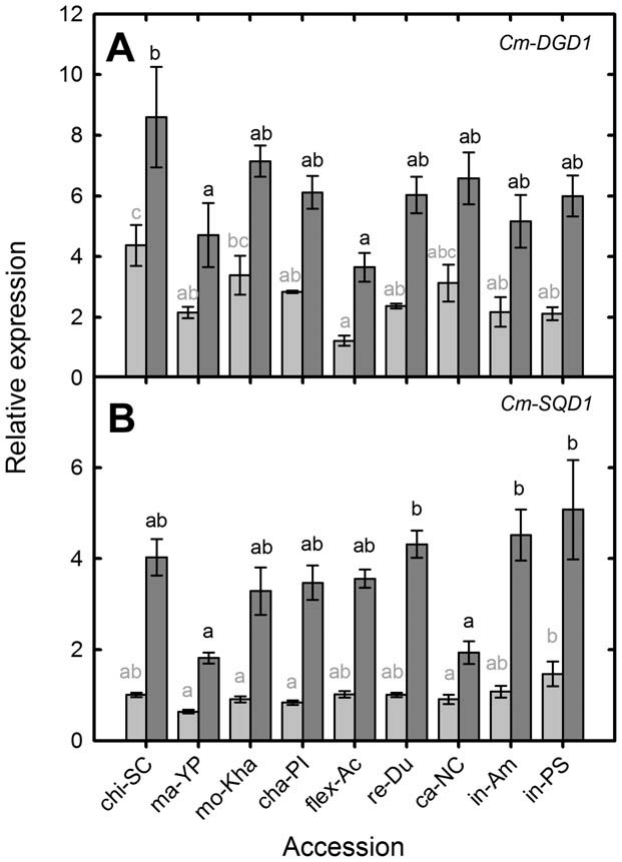
Relative root transcript abundance for *Cm-DGD1*, (b) *Cm-SQD1* in nine different melon accessions under P replete and P starved conditions. Relative transcript abundance for (a) *Cm-DGD1* [MU51583], (b) *Cm-SQD1* [MU52028] in different melon accessions grown hydroponically with a full nutrient solution (Control, light grey) or nutrient solution containing no phosphate (NoP, dark grey) for 21 d. Transcript abundance was measured using quantitative PCR (Q-PCR) and expressed relative to that of the housekeeping gene (*Cm-Ubiquitin*). Grey bars with the same grey letter are not significant different at *P*<0.05 by Newmans Keuls multiple range test. Dark grey bars with the same letter are not significant different at *P*<0.05 by Newmans Keuls multiple range test.

Genes involved in the manipulation of lipid membrane composition under Pi starvation showed increases in expression under NoP conditions relative to control conditions. The expression of *Cm-DGD1* (ICUGI unigen MU 51583), a putative digalactosyl diacyglycerol synthase, increased its expression approximately 2-fold under NoP conditions (Figure 6A). The accession with highest expression under NoP was chi-SC (8.6) and the lowest were ma-YP (4.71) and flex-Ac (3.65). *Cm-SQD1* (ICUGI unigene MU52028) encodes for a putative UDP sulfoquinovose synthase (SQD1) and was induced 2 to 5-fold under NoP conditions relative to control conditions (Figure 6B). Re-Du, in-Am and in-PS showed the greatest relative expression in NoP (4–5) and ma-Yp with ca-NC the lowest (2).

*Cm-DGD1* and *Cm-SQD1* changes in expression were positively correlated with PUtE, indicating that a higher increase in the expression of these genes may be involved in a higher internal P utilization. *Cm-DGD1* was also negative correlated with SFW, RFW, shoot P content and PPUE in control conditions. Indicating that high biomass and PUE accessions under control conditions, have lower basal levels of expression.

### P signaling

**Figure 7:**
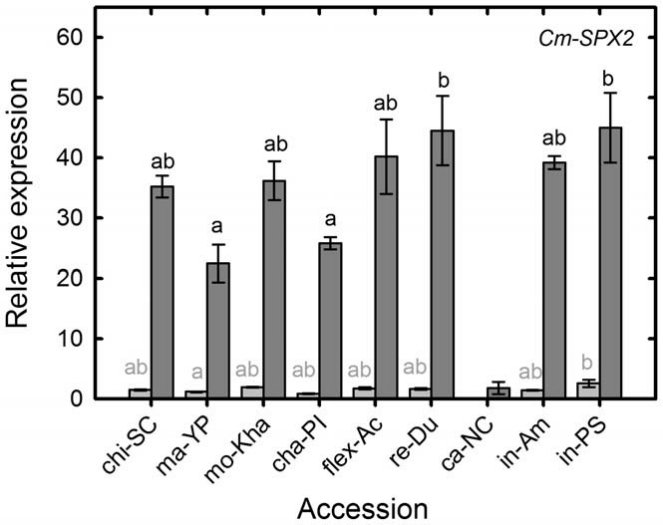
Relative root transcript abundance for *CmSPX2* in nine different melon accessions under P replete and P starved conditions. Relative transcript abundance for *CmSPX2* [MU43709/MU63649] in different melon accessions grown hydroponically with a full nutrient solution (Control, light grey) or nutrient solution containing no phosphate (NoP, dark grey) for 21 d. Transcript abundance was measured using quantitative PCR (Q-PCR) and expressed relative to that of the housekeeping gene (*CmUbiquitin*). Grey bars with the same grey letter are not significant different at *P*<0.05 by Newmans Keuls multiple range test. Dark grey bars with the same letter are not significant different at *P*<0.05 by Newmans Keuls multiple range test.

*Cm-SPX2* (ICUGI unigene MU43709) a putative transport protein with a SPX domain was impossible to correctly amplify ca-NC samples. Under NoP conditions, the expression of this gene was 44.5 times higher the basal expression (Figure 7). The higher change of expression was experienced by cha-PI which passed from a basal level of expression of 0.86 to an expression in NoP of 25.78, which in turn was one of the lowest levels of expression in comparison with other accessions. The higher levels of expression in NoP were reached by re-Du and in-PS 44.51 and 45 respectively (Figure 7).

Despite the expression of *Cm-SPX2* was the highest under NoP from all the studied genes, it showed no positive correlations with phenotypical traits, but had a negative correlation with RSRa in both control and NoP conditions.

## Discussion

### Biomass allocation and PUE traits vary significantly between melon accessions

The responses displayed by the nine melon accessions studied were diverse in terms of plant morphology, P uptake and use. Plants were evaluated in an early stage and, therefore, part of the observed variation might be due to differences in the seed size. Nevertheless, previous studies with these accessions have shown that seed weight affects the initial biomass, especially in NoP conditions, but not other parameters [31]. The studied accessions can be divided into three groups; the first group is composed of chi-SC, ma-YP and ca-NC, which were small plants and had low measures of PUE, but high RSRa; the second group is composed of cha-PI, re-Du and in-PS, which had intermediate values for PUE; the third group is composed by mo-kha, flex-Ac and in-Am, which had greater biomass and higher values of PUE.

### Phosphate mobilization and re-mobilization

Transcriptional changes under Pi starvation have been demonstrated in various crop plants [10]–[17], [20], [37]–[44]. Phosphatases and ribonucleases, are important in the mobilization of the organic P in soil for root absorption and remobilization of organic P [19], [20], [26]. Our results demonstrate an increase in the expression of two purple acid phosphatases and a ribonuclease under Pi starvation in melon (Figure 4). PAPs have different roles in P response, some are secreted and some have internal functions [9], [45]. Both melon transcripts studied, *Cm-PAP10.1* and *Cm-PAP10.2*, showed the highest homology to *AtPAP10* (At2g16430.2), but they displayed different responses to Pi starvation and different pattern of correlation with other traits, suggesting different roles in Pi nutrition. The fact that *Cm-PAP10.1* was associated with improved biomass accumulation and PUE of accessions in control and not under NoP conditions may suggest that it could be a secreted acid phosphatase. Under NoP conditions, the lack of available Pi or organic sources of P in the hydroponic system would not result in improved acquisition as a consequence of increased expression/secretion of this PAP. In contrast, the correlation between *Cm-PAP10.2* expression and PUE, indicate that it could be an internal PAP involved in P efficiency remobilizing Pi under NoP conditions (Table 2). Induction of *Cm-RNS1* expression was very intense under NoP conditions (Figure 4C). The orthologue of *AtRNS1*, encoding a secreted protein up-regulated during Pi starvation, it has been suggested to be involved in Pi mobilization from RNA sources extracellular and intracellular under Pi stress, senescence and wounding [19] [10]. Our correlation results may support a similar role for the melon orthologue described here, but further analysis of RNS and PAPs in melon must be undertaken to define their role in Pi starvation.

### Metabolism

Several genes related to glycolysis increase their expression under Pi starvation, increasing the efficiency of this biochemical pathway in terms of Pi [11], [21], [22]. *Cm-PPCK* is an orthologue of *AtPPCK1* (At1g08650.1). In arabidopsis PPCK genes are rapidly induced by Pi starvation leading to increase phosphorylation of phosphenolpyruvate carboxylase (PEPC). PEPC bypasses the pyruvate kinase step in glycolysis, and the phosphorylation of PEPC reduces its sensitivity to malate inhibition [46]. Increases in the concentration of PPCK have also been reported to be involved in induction of synthesis and exudation organic acids. Anthocyanin accumulation is a well-documented response to Pi starvation [41], and it is suggested to protect plants against oxidative stress [47]. As expected our transcript *Cm-transferase*, which is an orthologue of the arabidopsis anthocyanin 5-aromatic acetyltransferase gene (At5g39090.1) was upregulated under Pi starvation (Figure 5B). We found differences in the expression of *Cm-PPCK* and *Cm-transferase* genes indicating a different level of response among accessions. These responses were just moderately correlated with P use and efficiency parameters.

### Alteration membrane lipid composition and metabolism

Under Pi starvation lipid composition of plant membranes changes drastically, decreasing phospholipids and increasing non-phosphorous lipids [23]–[26]. In this experiment, we monitored the expression of putative orthologous genes for UDP-sulfoquinovose synthase/SQD1 (At4g33030.1) and UDP-galactosyltransferase/DGD1 (At3g11670.1). Both genes were upregulated under NoP conditions, and as with other genes monitored here there were differences in the expression levels among accessions, although they were less intense than for other genes (Figure 6). Nevertheless there was a moderate positive correlation among the best performing accessions between PUtE and expression of *Cm-SQD1* and *Cm-DGD1*.

### P sensing and transport

SPX domain containing proteins are involved in Pi transport and sensing [48], [49]. The arabidopsis genome contains 20 genes encoding SPX-domain proteins. Our transcript *Cm-SPX2* is orthologue of *AtSPX2* (At2g26660.1), At-SPX2 isoform, is targeted to the nucleus and is weakly induced by Pi starvation [50]. However, it is difficult to drawn inferences about the functional equivalence, if any, among the SPX isoforms between melon and arabidopsis. The *At-PHO1* gene (At3g23430) encodes one SPX protein that is involved in the regulation of Pi homeostasis through the Pi loading to the xylem [48]. In the case of *Cm-SPX2*, it the highest level of induction among the genes studied (Figure 7), but no correlations between its expression and phenotypical traits was observed (Table 2). This may indicate a general response of all the accessions studied despite the different levels of expression.

### Conclusion

The response of plants to Pi starvation is complex involving many genes. Explaining the diversity of Pi starvation responses for different species accessions using nine genes didn't fully account for the phenotypic diversity observed. However, the expression profiles of nine Pi starvation responsive genes in melon have been demonstrated, as well as their differential response among nine diverse accessions. Accessions with higher measures of PUE, such Flex-Ac, in-Am and mo-kha, also had higher *Cm-PAP10.1*, *Cm-PAP10.2* and *Cm-RNS1* expression in NoP than any other accessions, whereas the accessions with lower measures of PUE (chi-SC, ma-YP and ca-NC) had low expression levels for these genes. Therefore higher mobilization and remobilization of P may be a preferential source of diversity in Pi starvation adaptation in melon accessions. The response of other putative Pi responsive genes was also demonstrated to be accession dependent, but they collectively had weaker correlations with measures of biomass accumulation and measures of PUE. Further investigations are required to assess the biochemical impacts of these gene expression changes on their substrates and to characterize additional orthologues of Pi starvation inducible genes for correlations with PUE traits, in the light of new sequence data for melon and related species.

## Materials and Methods

### Plant material

Nine melon accessions were used in this study. They belong to 5 botanical varieties of both subspecies; three to ssp. *agrestis*: two *conomon* from Japan (chi-SC, and ma-YP), and one *momordica* from India (mo-kha); and six to ssp. *melo*: two *flexuosus* types (cha-PI and flex-Ac), and four varieties of the main commercial groups, *reticulatus* (re-Du), *cantalupensis* (ca-NC) and two *inodorus*, Piel de sapo and Amarillo (In-Ps and In-Am; Table 1). These accessions were selected in a previous study as they represent different responses to P starvation in the species [31].

### Experimental design

Seeds were germinated on germination paper for 6 d and then transferred to 34 L tanks for hydroponic growth. There were two different treatments with three plants per accession grown in each treatment: i) control nutrient solution (C), composed of: 3 mM KNO_3_, 2 mM Ca(NO_3_)_2_•4H_2_O, 0.5 mM MgSO_4_•7H_2_O, 0.5 mM (NH_4_)H_2_PO_4_, 25 µM KCl, 12.5 µM H_3_BO_3_, 1 µM MnSO_4_•H2O, 1 µM ZnSO_4_•7H_2_O, 0.25 µM CuSO_4_•5H2O, 1.3 µM (NH_4_)_6_MO_7_O_24_•4H_2_O, and 25 µM Fe-NaEDTA; ii) nutrient solution deficient in Pi (NoP) in which 0.5 mM (NH_4_)_2_SO_4_ was added instead of (NH_4_)H_2_PO_4_ [31]. The experiment was repeated three independent times.

Plants were grown in a glasshouse compartment at Warwick HRI latitude 52°12′31″N, longitude 1°36′06″W, 46 m above sea level with 16 h light, 26°C day and 20°C night and 60% humidity. Twenty-one days after sowing, plants were harvested and fresh weight of roots and shoots was measured (RFW and SFW respectively) and the root to shoot ratio calculated as RFW/SFW (RSRa). The length of the longest root of the plant was measured (RL). Cotyledons were removed and shoots were oven dried for total P quantification, whereas roots were frozen with liquid nitrogen for RNA extraction.

### Total P determination

Shoot dry weight (SDW) was recorded after oven-drying at 60°C for 72 h. Shoot samples were digested by the addition of 2 mL nitric acid to 0.3 g dried, ground material and processed in a closed vessel acid digestion microwave (MARSXpress; CEM Corporation, Matthews, NC, USA). Digested samples were diluted with 23 mL of de-ionised water and analysed using inductively-coupled plasma emission spectrometry (ICP-ES; JY Ultima 2, Jobin Yvon Ltd., Stanmore, Middlesex, UK) to determine shoot P concentrations.

Using the shoot dry weight values and the P concentration in the tissues shoot-[P], several measurements of P-use efficiency (PUE) were calculated [51]:

Total amount of P in the shoot:

Shoot-P (mg P plant^−1^) = [P]* SDW

P-uptake efficiency (PUpE) was calculated as the increase in total P content:

PUpE (mg P) = ([P_C_]*SDW_C_)−([P_NoP_]*SDW_NoP_)

Phosphorus utilization efficiency (PUtE) was calculated as the increase in yield per unit increase in P content:

PUtE (mg SDW mg^−1^ P) = (SDW_C_−SDW_NoP_)/[([P_C_]*SDW_C_)−([P_NoP_]*SDW_NoP_)]

Physiological P-use efficiency (PPUE) was calculated as the yield divided by tissue P concentration for either the C or NoP treatment:

PPUE (mg^2^ SDW mg^−1^ P) = SDW_C_/[P_C_] or SDW_NoP_/[P_NoP_]

Where SDW is the shoot dry weight for either control (C), or treatments (NoP) and [P] is the tissue P concentration for either control (C), or P starving treatments (NoP).

### RNA extraction and reverse transcription

Total RNA was extracted from frozen root tissue using the TRIzol method as described in [10]. To each sample, 1 mL of TRIzol reagent was added, and total RNA was subsequently extracted according to the manufacturer's instructions (Invitrogen, Paisley, UK), with the following modifications: (i) after homogenization with the TRIzol reagent, the samples were centrifuged to remove any remaining plant material and the supernatant was then transferred to a clean Eppendorf tube, and (ii) to aid precipitation of RNA from the aqueous phase, 0.25 mL of isopropanol and 0.25 mL of 1.2 M NaCl solution containing 0.8 M sodium citrate were added. This procedure precipitated the RNA whilst maintaining the proteoglycans and polysaccharides in a soluble form. Extracted total RNA was then purified using the ‘RNA Cleanup’ protocol for RNeasy columns (Qiagen, Hilden, Germany). RNA yield and purity were determined using a NanoDrop spectrophotometer (Thermo Fisher Scientific Inc, Waltham, MA) and agarose gels.

Reverse transcription was performed on 1 µg of total RNA using the ThermoScript RT-PCR system (Invitrogen, Paisley, UK). The cDNA synthesis reaction was carried out using 0.2 µL random hexamers (50 ng µL^−1^) and 0.8 µL Oligo(dT)_20_ primer (50 µM) according to the manufacturer's instructions.

### Gene identification and primer design

The expression of selected genes, involved in response to P starvation was analyzed by quantitative PCR (Q-PCR). The melon orthologues of genes that are known to be up- or down-regulated under P starvation in *A. thaliana* (Table 3) were identified *in silico* using available melon sequence data ([33], [35]. BLASTx was used to screen the ICUGI melon EST collection [34].

**Table 3:** List of genes analyzed and their function.

Melon Gene	unigene^a^	Primers	Description
**Phosphate mobilization**			
*Cm-PAP10.1*	MU46092	aacattctggtttgtcactcctc	AT2G16430.2 PAP10 (Purple acid phosphatase 10); acid phosphatase/protein serine/threonine phosphatase
		tatgcgggtttcgttcgtag	
*Cm-PAP10.2*	MU50216	catggtcggtcctgatgttc	AT2G16430.2 PAP10 (Purple acid phosphatase 10); acid phosphatase/protein serine/threonine phosphatase
		tgattgtccgcgtaagaaag	
*Cm-RNS1*	MU47003	gacaggaaaaccaagtgctg	AT2G02990.1 RNS1 (Ribonuclease 1); endoribonuclease/ribonuclease
		tctccatactgctcaccaaatc	
**Carbon and secondary metabolism**			
*Cm-PPCK1*	MU52466	cgatgaaactgacaaggaatg	AT1G08650.1 PPCK1 (Phosphoenolpyruvate carboxylase kinase); kinase/protein serine/threonine kinase
		ggtcggaaatcaaacagagg	
*Cm-transferase*	MU47437	ctttgatttgatgtggctaagg	AT5G39090.1 transferase family protein, similar to anthocyanin 5-aromatic acetyltransferase
		ccagaggaagataatgacgaag	
**Alteration membrane lipid composition and metabolism**			
*Cm-DGD1*	MU51583	tcgtaaatggcttgaggaaag	AT3G11670.1 DGD1 (Digalactosyl diacylglycerol deficient 1); UDP-galactosyltransferase/galactolipid galactosyltransferase/transferase, transferring glycosyl groups
		ttggaaggaacaaactgagaag	
*Cm-SQD1*	MU52028	ccgcacttcatgtttctcag	AT4G33030.1 SQD1; UDPsulfoquinovose synthase/sulfotransferase
		gccatatcaacgtgttccac	
**P sensing and transport**			
*Cm-SPX2*	MU43709	gcgagatggttttgttggag	AT2G26660.1 SPX2 (SPX domain gene 2) similar to PHO1 (At-SPX2)
	MU63649	tctgtggtgaagaagggttg	
**Control gene**			
*Cm-Ubiquiti*n	MU45991	tgtttctaaggtgctgttgtcc	Ubiquitin carrier ligase protein
		cgtgctgttgcttcatacttg	

a Sequences available at ICUGI [34].

Primers for quantitative PCR were designed across exon boundaries of the candidate genes with the Primer3, and hairpins and dimer formation were checked with PrimerSelect (DNASTAR, Inc., Madison, WI). Primers were also designed for six possible housekeeping genes. The stability of the housekeeping genes was evaluated with samples of all accessions and both treatments with the program GeNorm (http://medgen.ugent.be/~jvdesomp/genorm/). The ubiquitin gene (MU45991) was selected as housekeeping gene because of its stability and good dissociation curve. Primers used for amplifying the selected target genes are listed in Table 3.

### Gene expression analysis by quantitative RT-PCR and statistical analysis

Analysis of transcript levels of each gene was determined by quantitative PCR using an ABI Prism 7900 HT (Applied Biosystems, Paisley, UK sequence detector and SYBR Green fluorescent dye (Bioline Ltd., London, UK). For each accession and treatment, cDNA samples from three biological replicates used. Intra-assay variation was evaluated by performing all amplification reactions in technical triplicate. Reactions (10 µL volume) were conducted in 384-well plates consisting of 2-ng cDNA sample, 10 µM forward and reverse primer, and 5 µL of 2× SYBR Green PCR master mix (Bioline Ltd). The quantitative PCR reaction conditions were 50°C (2 min) followed by 95°C (10 min) for 1 cycle, then 95°C (15 s) followed by 60°C (1 min) for 40 cycles. This was followed by a dissociation step of 95°C for 15 s, 60°C for 15 s and 95°C for 15 s. The dissociation step was included to generate data for melting curve analysis so that primer dimers or nonspecific products could be detected in the reaction. A control reaction of each sample was included in each plate using 2 ng of total RNA, to assess the presence of genomic contamination and primer dimers and/or primer contamination

The cycle threshold and normalized fluorescence (ΔR_n_) values were determined for each sample during the quantitative PCR cycling reaction using the ABI Prism sequence detector software (v2.0). The cycle threshold value was calculated using a threshold value set at 0.2. A four point standard curve was constructed from 10-fold dilutions of cDNA for each specific gene and the housekeeping gene. Efficiency was calculated from the slope of the linear correlation between Cp (crossing points) values of each dilution and the logarithm of the corresponding amount of RNA, according to the equation 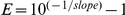. Since efficiency for all genes ranged within 95–105, the relative expression of target genes was related to the expression of the housekeeping gene (*Cm-Ubiquitin*) with the equation 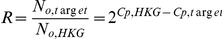


Pearson correlations among traits and ANOVA analyses were performed using Statgraphics 5.1 software, Newmans-Keuls multiple Range test was used to separate main effect means when the F-test was significant.
